# Methodological considerations for evaluating scale-up programmes in healthcare: a methods review

**DOI:** 10.1093/intqhc/mzaf120

**Published:** 2025-12-05

**Authors:** Bastiaan Van Grootven, Serena Sibilio, Nereide Curreri, Jianan Huang, Laurie Corna, Nathalie I H Wellens, Franziska Zúñiga

**Affiliations:** Nursing Science, Department of Public Health, University of Basel, Basel, Switzerland; Department of Public Health and Primary Care, KU Leuven, Leuven, Belgium; Nursing Science, Department of Public Health, University of Basel, Basel, Switzerland; Department of Business Economics, Health and Social Care, University of Applied Sciences and Arts of Southern Switzerland, Manno, Switzerland; Nursing Science, Department of Public Health, University of Basel, Basel, Switzerland; Department of Business Economics, Health and Social Care, University of Applied Sciences and Arts of Southern Switzerland, Manno, Switzerland; La Source School of Nursing, HES-SO University of Applied Sciences and Arts Western Switzerland, Lausanne, Switzerland; Nursing Science, Department of Public Health, University of Basel, Basel, Switzerland

**Keywords:** public health, healthcare system, Implementation science, Scale-up, general methodology

## Abstract

**Background:**

The ultimate goal of many research projects is to achieve sustained improvements in health outcomes at population level. Scale-up refers to the integration of an effective intervention in routine practice and policy. Pertinent questions pertain to the appropriate study design in evaluating scale-up success.

**Methods:**

A methodological review was conducted to determine how the scale-up of evidence-based interventions in healthcare can be evaluated. Specifically, we examined (i) appropriate research designs; (ii) outcomes and measures, endpoints; and (iii) key methodological considerations. Databases were searched and supplemented with hand searching journals and screening references and prospective citations. A narrative synthesis of included studies was produced.

**Results:**

Several pre-scale-up design considerations were identified, including the need to assess the strength of the evidence base, develop a programme theory to guide measurements, and conduct a contextual analysis to inform implementation determinants. Evaluating baseline performance was recommended to define improvement targets, while scalability assessments were advocated to evaluate whether the intervention can be expanded with success. For scale-up evaluation, multiple evaluation domains and design considerations were identified. Adoption was described as the intention, decision, or action to try an intervention, which can be surveyed and supplemented with interviews to understand adoption decisions and processes. Coverage and reach were used to assess expansion of scale-up, defined respectively as the proportion of organizations implementing and the proportion of the target population reached, ideally tracked longitudinally with predefined targets. Routinely collected information, including insurance/billing data or administrative data, and survey designs can be used. Institutionalization referred to the integration of interventions into existing systems and structures. Recommended methods included stakeholder interviews, policy document reviews, and surveys with implementers, providers, and policymakers. Costs related to scale-up go beyond direct implementation costs and included broader system costs and costs associated with institutionalization. Evaluating effectiveness was considered important, including exploring variation across subgroups, providers, and regions. Design recommendations included predominantly nonexperimental designs, using pre–post and time series designs. Monitoring fidelity and adaptations were advocated, e.g. using observations. Across domains, mixed-methods approaches were recommended to capture both outcomes and the mechanisms through which they were achieved, recognizing scale-up as an iterative and adaptive process.

**Conclusion:**

Longitudinal, adaptive, and mixed methods designs are needed to capture real-world implementation dynamics. We propose an initial conceptualization of scale-up success, defined by the main dimensions of coverage, reach, effectiveness, and institutionalization, which are contingent on cost of scale-up, adoption, fidelity, and adaptations.

## Introduction

The ultimate goal is to achieve sustained improvements in health outcomes at population level through scalable interventions [1]. In this study, we use the term evidence-based intervention to refer to the clinical or public health practice itself, e.g. a drug or exercise protocol (‘the thing’), that has demonstrated effectiveness. Achieving population-level impact requires, first, generating evidence on the effectiveness of this intervention, and second, generating evidence on how best to implement it in real-world settings (‘how to do the thing’—the implementation) [2]. Implementation science bridges the gap between research and practice by systematically studying how evidence-based interventions can be integrated, adopted, and sustained in real-world settings [3, 4].

Implementation typically begins within local health systems, where an implementation team plays a central role in introducing an evidence-based intervention. Implementation teams often establish partnerships, conduct a contextual analysis, deliver education and training, conduct audits with feedback, and facilitate organizational changes to support the adoption of the innovation [5, 6]. This is done through a planned, often linear, approach to overcome barriers and harnessing facilitators in the system [7]. Once the implementation project has ended, both the sustainability and the expansion towards larger regions are challenging [8]. In particular, the initial implementation study may be difficult to replicate at scale if assumptions around resources and delivery mechanisms are no longer feasible. Contextual factors are often a key barrier (e.g. lack of structural funding to continue the implementation, implementation climate) [9]. Likewise, scaling-up evidence-based interventions necessitate overcoming contextual barriers to offer interventions to a larger group of patients or settings.

Scale-up refers to the integration of an effective intervention in routine practice and policy [10]. Scale-up requires a shift from local implementation, often supported by high levels of external research involvement, to a sustainable model where systems own and adopt the evidence-based intervention components to the level that they become the new ‘usual care’ [11]. It involves strategic considerations related to human resources, infrastructure, financing, education, and may therefore require policy change and/or policy support [12]. Scaling-up expands the implementation challenge to address variations in the motivation of adopters, resource availability, and system readiness across broader and potentially less-receptive providers [12]. As a consequence, an implementation plan that worked well in one setting may not work to the same degree in another [7].

The complexity of scaling-up may also extend to the design of scale-up evaluation studies. In comparison with study designs and methods related to (comparative) effectiveness research, the design of studies evaluating scale-up of evidence-based interventions has received little attention. By its nature, scale-up implies less control by the research team and larger samples, which may not fit well-established research designs like a randomized controlled trial. Investigating sustainability at scale may be another scenario where traditional evaluation design may not be optimal. For example, studying variation in patient or system outcomes over time might be more informative for sustainability than a traditional between-group comparison [13].

Pertinent questions relate to the appropriate study design in evaluating scale-up success. Overall, guidance is missing on which study design is appropriate to evaluate scale-up and which outcomes could be used to measure the success of scale-up of healthcare interventions. We therefore conducted a methods review to determine how the scale-up of evidence-based interventions in healthcare settings can be evaluated. Specifically, the study aims to: (i) determine which research designs are appropriate for evaluating interventions being scaled-up; (ii) determine which types of (implementation) outcomes, which outcome measures, and which endpoints are appropriate; and (iii) identify methodological considerations that are relevant. Note that the review does not address how interventions should be scaled-up.

## Methods

A protocol was drafted based on the adaptation of the PRISMA guidelines for the reporting of meta-epidemiological methodology research [14]. The three aims are reported in one integrated review report.

### Eligibility criteria

Studies were eligible for inclusion based on the three key objectives related to evaluating the scale-up of evidence-based interventions in healthcare. For the purpose of this review, scale-up refers to the integration of an effective intervention in routine practice [10]. Research designs: studies that provide methodological advice on external and internal validity, or compare the validity of multiple research designs, in the context of scaling up interventions. Outcomes and measurements: studies that offer guidance on or test the validity of outcomes, outcome measurements, or endpoints relevant to scale-up. Methodological considerations: studies that discuss methodological challenges or provide advice specific to scaling up. A study needed to address at least one of these objectives to be considered for inclusion. Studies that focused on programme evaluation, without specifically considering scaling up evidence-based interventions, were excluded.

### Information sources

Two bibliographic databases, PubMed and Embase, were searched from inception until January 2024. In addition, journals related to implementation science and meta-epidemiology were hand searched. We included hand searching as additional strategy, because we anticipated that methods papers would be poorly indexed in databases. These included the following journals: *International Journal for Quality in Health Care*, *Implementation Science*, *Implementation Science Communications*, *Implementation Research and Practice*, *BMJ Quality and Safety*, *Administration and Policy in Mental Health and Mental Health Services Research*, *BMC Health Services Research*, *Lancet Public Health*, *Milbank Quarterly*, *International Journal of Epidemiology*, *European Journal of Epidemiology*, *Journal of Clinical Epidemiology*, and *BMC Medical Research Methodology*. Reference lists and prospective citations of included studies, and names of authors who published on the topic were additionally searched.

### Search strategy

The search strategy utilized a combination of MeSH/Emtree terms and free-text keywords to identify relevant studies up to February 2024. No filters were applied. For Objective 1 (appropriate research designs), the search combined terms related to scale-up (e.g. ‘scale-up’, ‘scale up’, ‘scaling-up’, ‘scaling up’, ‘system implementation’) and methodological aspects (e.g. ‘internal validity’, ‘external validity’, ‘epidemiological research’, ‘meta-epidemiology’, ‘bias’, ‘validity’, ‘guidance’). For Objective 2 (outcomes and measurements), terms targeted implementation outcomes (e.g. ‘implementation outcome*’, ‘outcome*’) in the context of scale-up, along with ‘review’ and ‘literature’. For Objective 3 (methodological considerations), the search focused on scale-up and broader guidance or methodological advice (e.g. ‘guidance’, ‘methodological advice’). Hand searches in selected journals included simple keywords ‘scale-up’ and ‘scaling-up’. Studies identified under one objective but providing information relevant to another were reassigned as appropriate to ensure comprehensive coverage across all objectives.

### Selection process

References were uploaded in Endnote, and duplicates were removed. Studies were selected in a two-step screening process, i.e. first screening title and abstract, and then evaluating full-text articles of potentially eligible studies. One researcher conducted the search strategy, and a second researcher verified the selection of full-text articles. One meeting was organized to find consensus on the final selection of included articles. The selection process was documented in Endnote.

### Data collection and synthesis method

Data were documented in an Excel database by one researcher and verified by a second researcher. The synthesis was jointly discussed. One researcher produced a narrative description of the study results. The results were reported in a narrative text.

## Results

A total of 6338 titles and abstracts were screened after the database searches. A total of 88 full-text articles were assessed for eligibility. For Objective 1, six studies were included [15–20]. For Objective 2, 11 studies were included [12, 17, 18, 20–27]. For Objective 3, 10 studies were included [12, 16, 17, 19, 20, 28–32]. A total of 31 additional full-text articles were assessed for eligibility based on the additional search strategies, which resulted in three additional inclusions. Included articles spanned different disciplines, including behavioural science (*n* = 3) [18, 23, 31], implementation science (*n* = 7) [19–22, 24, 28, 32], public health (*n* = 3) [12, 25, 29], and safety and quality assurance (*n* = 3) [16, 17, 27]. First, pre-scale-up considerations are reported. Then, outcomes that were reported in scale-up literature are reported. Per outcome, design considerations are reported.

### Pre-scale-up considerations

The starting point of scale-up is the availability of an effective intervention. A systematic review can be conducted to assess the evidence, i.e. determine how likely the observed effects are to be reliable and applicable across different contexts [18, 19]. Furthermore, the evidence base will inform the need to further evaluate effectiveness, guiding the choice of an appropriate study design. A dedicated programme theory and scale-up plan will be needed. The programme theory should outline how the evidence-based intervention is expected to achieve outcomes at scale, identify contextual factors influencing scale-up, and clarify what intervention components may need to be adapted [21]. This information can be used to define specific indicators for measurement [16]. The programme theory ideally builds on a contextual analysis, which will help identify implementation determinants, guide the scale-up plan, and inform the measurement of contextual variables to be included in the scale-up evaluation [16, 17, 19]. Qualitative methods, e.g. ethnography, are suited for this [17]. Quantitative methods can be used to identify the baseline performance of organizations and inform scale-up targets that indicate success, which should be defined explicitly [12]. Additionally, exploring variation in baseline performance may help inform a sampling strategy for the contextual analysis, identifying lower-higher performing organizations, and creating richer understanding of implementation determinants [17]. Lastly, a scalability assessment can be conducted to evaluate the interventions’ potential for adoption, spread, and sustainability while retaining effectiveness. Dedicated scalability assessment tools are available for this [28] (see Table 1 for an overview).

**Table 1.** Pre-scale up considerations

Domain	Considerations	Potential methods or tools
Evidence base	Assess the strength, consistency, and transferability of the evidence for effectiveness of an intervention under consideration for scale-up. For scale-up, it is likely important to explore subgroup effects and differences in context to understand potential variations in intervention effectiveness across populations and settings. This will inform the need to embed further effectiveness evaluations in the scale-up. Evaluator may need to reflect on differences in evidence from clinical trials and pragmatic trials. High GRADE evidence from clinical trials may not translate well to impact on practice, when conditions in the clinical trial differ to much from practice. Pragmatic trials are expected to align more with the context of daily practice.	Systematic review. Subgroup analysis or meta-regression can be used to explore variation in effects.
Programme theory	A programme theory clarifies the mechanisms by which the evidence-based intervention is expected to produce outcomes and the contextual factors that may influence them, thereby guiding the selection of process, outcome, and context measures to be included in a scale-up evaluation study.	Theory of change, logic models
Contextual analysis	Examine the environment in which the scale-up will take place to identify potential implementation determinants. The developed programme theory can be used to guide the interview guide or observations in a qualitative exploration, and be updated with new contextual insights.	Stakeholder mapping, qualitative interviews, ethnography. If resources are scarce, rapid ethnography can be considered
Baseline performance	Quantify current performance in the intended scale-up setting to set realistic targets.	Analysis of routinely (automated) collected data, surveys, cross-sectional study
Scalability assessment	A scalability assessment examines whether an effective intervention can be implemented and sustained at a larger scale without losing its benefits. This helps assess readiness, resource requirements, equity, and fidelity versus adaptation needs. This process supports decisions on whether and how to proceed with scale-up.	Scalability assessment tools

### Design considerations to evaluate scale-up

Scale-up is generally considered a long-term and multiphased process [11]. Consequently, multiple methods, both quantitative and qualitative, using longitudinal measurements within an adaptive evaluation framework are advocated (see Table 2 for an overview of evaluation domains) [12, 17]. Randomized controlled trials are generally not recommended as they often include unrealistic conditions too far removed from real-world practice. A strict control group withholding effective care would be unethical, and challenges may exist in randomizing the exposure of interest [15, 20]. For example, randomization may not be feasible when the intervention is related to policy. Pragmatic trials using a stepped wedge design may overcome some of these issues, as all organizations will be exposed to the implementation over time [20]. In general, scale-up favours adaptive designs, for which other designs are recommended. Pre–post studies are indicated as they more closely align with real-world implementation contexts [18]. A critical element may be the consideration of the timescale in which outcomes are measured. There can be a considerable time lag between the launch of a scale-up, exposure to the implementation, the decision to try the intervention, and its delivery to a sufficiently large group of consumers. This can challenge causal inference when study time frames are long [17]. Monitoring influencing factors may be needed, and a difference-in-difference design could be considered. A time series design could also be considered, and can be particularly efficient when evaluators have access to data through registries or administrative data [19]. Embedding qualitative designs in the evaluation can be useful to investigate experiences with the implementation process, and study how organizations have adapted the intervention to their context. In particular, case studies can be considered to explore variation in implementation [17]. This mixed-methods approach will support an adaptive evaluation design where scale-up monitoring can inform adaptations to the scale-up plan, and improve the likelihood of success [17]. A scale-up could also focus on one region—evaluate, learn, adapt, and then move to the next region—or on spreading through providers or health systems [12, 21, 24].

**Table 2.** Scale-up evaluation domains^a^

Outcome	Measurement	Methodological considerations
Adoption	Number or proportion of organizations that agree to adopt the intervention.	Predicted by acceptability, feasibility, and appropriateness of the intervention within context Surveys can be used to inquire into adoption behaviour Longitudinal measurements are needed to capture adoption patterns over time Interviews or focus groups can provide insights into reasons for adoption or nonadoption
Reach	Number of proportion of the target population receiving the intervention.	Routinely collected administrative and/or clinical data can be useful sources Can vary across regions and subgroups; monitoring over time is critical Nonlinear process: gradual uptake may necessitate repeated measurement
Coverage	Proportion of targeted institutions implementing the intervention. Distribution across regions and providers can be relevant.	Extends adoption to system-level spread across organizations Definition of coverage ideally include predefined targets by region, provider, and timepoints. Study designs may need to account for dynamic coverage targets that evolve from regional to national scale-up.
Institutionalization	Structural indicators such as updated policies, education and training programmes, monitoring mechanisms, or changes in legal and financial frameworks.	Marks transition from externally supported to internally resourced delivery Assess system-level changes supporting sustained implementation Indicators include policies, guidelines, training, monitoring, health IT integration, and budget al.ocation Methods may include stakeholder interviews, policy document reviews, and surveys with implementers, providers, and policymakers.
Cost of scale-up	Direct and indirect costs, including education, training, infrastructure, organizational restructuring, and new or increased administrative costs.	Goes beyond direct implementation costs (training, education, materials) Includes broader system costs: infrastructure, workforce expansion, organizational restructuring, and administration Costs of institutionalization (e.g. legal reforms, permanent financing streams) must be separated from temporary project costs
Effectiveness	Impact on target outcomes of interest. Comparison with impact observed in previous evaluation studies is advocated. Can be operationalized as the extent to which effectiveness at scale corresponds with effectiveness observed in evidence-base.	Evidence maturity informs whether additional effectiveness studies are needed Evaluating effectiveness is generally recommended Large drops in effectiveness at scale are a common risk Evaluations should consider variation in effectiveness across settings, subgroups, and providers Study designs should avoid withholding the intervention (e.g. using stepped-wedge) Interrupted time series analysis may be appropriate if longitudinal routine data are available Nonrandomized designs can be preferred due to their alignment with real-world practice Quality improvement methods can inform individual organizations how they were affected by the scale-up design
Fidelity	Degree to which core intervention components are implemented as planned.	Adjustments made by organizations to fit local context Mixed methods (logbooks, surveys, interviews, observations) to assess fidelity and adaptations Qualitative data (case studies, observations) provide insights into how/why adaptations are made
Adaptations	Modifications made to the intervention to fit local contexts.	

a An important consideration is whether the evaluation is conducted externally or embedded. External evaluations may be perceived as more independent but often provide limited opportunities for real-time learning. Embedded evaluations enable continuous feedback and support iterative adaptations, albeit at the cost of reduced perceived independence [16].

### Adoption

Adoption refers to the intention, decision, or action to try an evidence-based intervention [33], and is likely predicted by perceptions of acceptability, feasibility, and appropriateness of the evidence-based intervention within the organization’s specific context [24]. Adoption can be measured as the number or proportion of organizations that agree to adopt the evidence-based intervention. Adoption is ideally monitored repeatedly over time, because system-level changes such as workforce capacity, financing, and policy mandates require time to become embedded into routine practice [17]. Evidence from early local or regional scale-up may be necessary to secure system-wide investments, which in turn can drive adoption decisions. Survey methodology could be used to inquire whether the intervention of interest has been implemented, or whether organizations report the intention to implement it in the future. It is equally important to understand why organizations choose to adopt, or not to adopt, an intervention [17]. Interviews or focus groups with stakeholders and implementers can provide insights into why organizations adopt, delay, or reject an intervention, and how contextual factors shape these decisions [23]. Interviews with healthcare professionals can extend these insights by examining how individuals within organizations anticipate adopting and adapting their own practices.

### Reach and coverage

Adoption will drive reach and coverage as more organizations implement the intervention. Reach can be seen as the number or proportion of persons in the target population who received the intervention [33]. The literature also uses the term coverage to refer to how many of the target organizations have the intervention implemented, divided by the total number of targeted organizations in the scale-up [21]. Ideally, this definition includes predefined targets for the coverage, which can further be specified by geographic area, or providers, with predefined timepoints (i.e. at what time after scale-up is coverage measured) [12]. Depending on the design of a scale-up study, the coverage targets could change over time, moving from regional scale-up to a multiregional or national scale-up [11]. Routinely collected information could further be used, including healthcare insurance billing data, public reporting data, or administrative information, to study the reach of an intervention in a population. Considering that scale-up is not likely to be a linear process and may take a significant amount of time, longitudinal measurements would be needed to evaluate evolution in coverage over time.

### Institutionalization

Scale-up will affect legal, political, and organizational structures to support and promote the adoption, and marks the transition from externally supported implementation to fully integrated service delivery [12, 27]. Institutionalization indicators could be defined to track how successfully the intervention is being integrated in the healthcare system and sustained through resources and policies without reliance on external support or project-based funding [12, 27]. Progression towards institutionalization could be monitored through structure indicators, such as the availability of policy, nomenclature, guidelines, education and training programmes, monitoring mechanisms, integration into health information systems, and dedicated budget lines [34]. In this way, institutionalization can be perceived to moderate adoption behaviour by creating a more favourable context. This could be seen as a process evaluation to evaluate whether the necessary contextual changes are in place [35]. Suggested methods include document and policy review, completed by interviews or a survey with stakeholders, implementers, providers, or policy members [12].

### Costs of scaling-up

Several sources refer to the importance of evaluating costs of scale-up [25]. While previous studies on effectiveness and small-scale implementation may include cost analyses, the cost of scaling up an intervention introduces additional complexities. Scale-up extends beyond direct implementation costs, as it often involves broader structural, operational, and system adaptations [12]. These include costs related to workforce training and education, changes in organizational processes, and the establishment of new administrative systems required for sustained delivery. Importantly, scale-up may also require additional investments to ensure alignment with existing policies, legal frameworks, and infrastructural support [36].

An essential component of scale-up cost analysis is the concept of cost of institutionalization [12, 36, 37]. Institutionalization may necessitate sustained funding commitments, advocacy efforts, and ongoing leadership support. Without a thorough understanding of these costs, scale-up initiatives risk not being financially sustainable, potentially leading to incomplete adoption or eventual abandonment of the intervention.

### Effectiveness

The choice to evaluate effectiveness may depend on the evidence base. Nonetheless, gathering effectiveness data during the scale-up process can promote adoption behaviour and spread of the scale-up to achieve a higher coverage [15, 21, 23]. The choice of whether and how to evaluate effectiveness may depend on the maturity of the evidence base, the availability of data relevant to specific contexts and subgroups, and the available resources within the evaluation team. Variation in effectiveness could be explored by type of region (rural–urban), for certain subgroups (with different risk profiles), or by type of provider. A comparative effectiveness study could also be introduced when problems with the scale-up are identified. The subsequent evaluation could then focus on studying the intervention versus a leaner version of the intervention to improve the adoptability of the intervention [30].

Effectiveness data can be supplemented with process indicators, which should be causally related to the outcome of interest [17]. This information can provide actionable feedback to individual organizations and help sustain the implementation. Quality improvement methods, such as statistical process control, can be used to monitor variation in their performance over time. For evaluators, examining organizational-level variation can highlight when aggregated effectiveness data may obscure important differences between organizations [17].

Scale-up will require monitoring of negative effects, because possible adverse events and inequalities are also scaled-up (e.g. lower uptake of digital health interventions among older persons in comparison to younger persons means that their access to certain services is not equal) [16].

### Fidelity and adaptations

Fidelity ensures that the core components of the intervention are delivered as intended, while adaptation allows necessary changes to fit local contexts [16, 21, 24]. Some have pointed out that fidelity may demand adaptations to the intervention [20]. Monitoring both processes is advocated, recognizing that scale-up requires balancing fidelity with context-specific tailoring to maintain effectiveness [19, 27]. Embedding this evaluation within implementation teams can support continuous monitoring of how interventions are implemented and adapted in practice, e.g. using audits or observation [16, 19].

## Discussion

### Statement of principal findings

**Figure 1:**
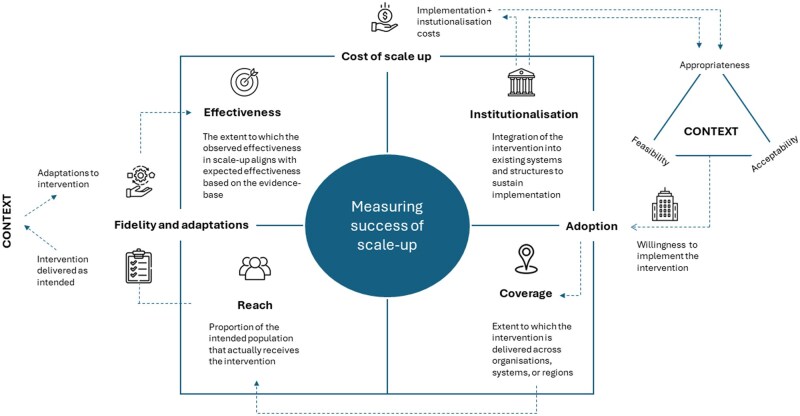
Conceptualization of measuring scale-up success. Measuring scale-up success can be framed as the ‘combined function of coverage, reach, and effectiveness, which is contingent on institutionalization’. This reflects whether an intervention is delivered widely, penetrates to the intended population, achieves its intended outcomes at scale, and is sustained through integration into existing systems and structures. The effectiveness dimension can be expressed as the extent to which the observed effectiveness in scale-up aligns with expected effectiveness based on the evidence base. Integration into the system will, among others, be determined by the cost of scaling-up. Institutionalization may help overcome implementation barriers and influence perceptions regarding appropriateness, acceptability, and feasibility, which in turn will influence adoption. This decision process will be different for organizations depending on their context. Similarly, costs associated with implementation may influence perceptions and adoption. As more organizations adopt the intervention, the coverage will increase, which results in an increase in reach of the intervention in the population. In persons who receive the intervention, the context will influence whether the intervention is delivered as designed, or if adaptations are made. If the fidelity to the core components of the intervention is sufficient, the effectiveness at scale is hypothesized to be similar to the effectiveness observed in the evidence base (assuming that the sample in the scale-up is sufficiently similar to those included in the evidence-base).

Our review underscores the need for comprehensive, often longitudinal and adaptive, evaluation designs using mixed methods that evaluate explicit improvement targets. Based on our findings, we propose a preliminary conceptualization of scale-up success, framed as the ‘combined function of coverage, reach, and effectiveness which is contingent on institutionalization’ (Fig. 1). This reflects whether an intervention is delivered widely, penetrates to the intended population, achieves its intended outcomes at scale, and is sustained through integration into existing systems and structures. The effectiveness dimension could be expressed as the extent to which the observed effectiveness in scale-up aligns with expected effectiveness based on the evidence base; significant drops, up to 50% in effectiveness between initial evidence and evidence from scale-up studies have been observed [38]. Integration into the system will, among others, be determined by the cost of scaling-up. Institutionalization will influence adoption decisions, while fidelity and adaptations will shape effectiveness, with both being influenced by the contextual fit of the intervention within the organization (both its inner and outer context) [39].

### Strengths and limitations

While multiple studies included methodological advice, these were smaller pieces of a large puzzle. It should be noted that multiple fields of evaluation exist, and that our review does not include an exhaustive review of all possible methods that could be used. For example, programme evaluation also includes evaluations of large public health programmes (that could be considered implemented at scale). The full-text study selection and data abstraction were reviewed by a second author. However, the title and abstract screening was performed by only one researcher. It is likely that not all methodological papers were identified, because of poor indexing and the diverse terminology used in this field. Nevertheless, the review synthesizes a substantial body of evidence to advance the methodological basis for scale-up evaluation. Lastly, it should be noted that no appraisal of study quality was performed. The focus is on summarizing methodological guidance, and not inferring results from empirical studies.

### Interpretation within the context of the wider literature

The results of our review do not entirely align with how scale-up studies have been evaluated. A systematic review of interventions in primary care observed that mostly outcomes at patient level were measured, reflecting health outcomes and delivery of care outcomes [21]. Only a minority measured coverage or institutionalization indicators. Most studies had used a quasi-experimental before-and-after study, with follow-up ranging from half a year to multiple years. A review conducted by our own team about scale-up in residential long-term care observed that most studies used a nonexperimental design and measured either outcome or process indicators of care [40]. While such designs are straightforward and often promoted to capture the real world, they may be prone to bias.

It appears that studies to date have mostly focussed on measuring patient outcomes. Hybrid designs were introduced in 2012 by Curran *et al.* [41] to study both effectiveness and implementation at the same time. Hybrid effectiveness-implementation designs have been used to study the scale-up of physical activity in the community. The programme ‘Choose to move’ used a hybrid type 2 design to study sustained effects on physical activity levels on the one hand, and the reach and fidelity on the other hand [39]. Similarly, Tsuzuki *et al.* [42] used a hybrid type 2 design and included reach, effectiveness, adoption, implementation, and maintenance as outcome measures. However, while such a design with multiple outcomes better reflects the methodological advice we described in this review, they are also more complex. In particular, concerns about multiple testing, sample size calculation, and defining a minimum clinically important difference, accounting for multiple outcomes (i.e. their joint effect) have been raised [43].

### Implications for policy, practice, and research

For policymakers, our results indicate the importance of setting measurable targets for scale-up and allocating resources beyond direct implementation, including changes at the infrastructure and system level. For implementation practitioners, our results point towards the importance of embedding evaluation targets in scale-up plans to monitor success and inform the need for adaptations. For researchers, our results highlight gaps in current methods used to evaluate scale-up. For example, developing more explicit standards and consensus on scale-up outcome measurement is likely to strengthen this field of research. We proposed an initial conceptualization for measuring success in scale-up.

## Conclusion

Our findings suggest that longitudinal, adaptive, and mixed-methods designs are needed to capture real-world implementation dynamics. We propose an initial conceptualization of scale-up success, defined by the main dimensions of coverage, reach, effectiveness, and institutionalization, which are contingent on cost of scale-up, adoption, fidelity, and adaptations. However, existing reviews of scale-up studies show that these domains are not yet well integrated into evaluations.

## Data Availability

The data underlying this article will be shared on reasonable request to the corresponding author.
